# Healthcare Data Breaches: Insights and Implications

**DOI:** 10.3390/healthcare8020133

**Published:** 2020-05-13

**Authors:** Adil Hussain Seh, Mohammad Zarour, Mamdouh Alenezi, Amal Krishna Sarkar, Alka Agrawal, Rajeev Kumar, Raees Ahmad Khan

**Affiliations:** 1 Department of Information Technology, Babasaheb Bhimrao Ambedkar University, Lucknow 226025, India; aadilhussain@gmail.com (A.H.S.); amalsarkar@gmail.com (A.K.S.); alka_csjmu@yahoo.co.in (A.A.); khanraees@yahoo.com (R.A.K.); 2 College of Computer & Information Sciences, Prince Sultan University, Riyadh 12435, Saudi Arabia; mzarour@psu.edu.sa (M.Z.); malenezi@psu.edu.sa (M.A.); 3 System Manager, Sanjay Gandhi Post Graduate Institute of Medical Sciences, Lucknow 226014, India

**Keywords:** healthcare data breaches, data confidentiality, data security, cost effectiveness, data analysis, time series analysis, data breach forecasting, cost forecasting

## Abstract

The Internet of Medical Things, Smart Devices, Information Systems, and Cloud Services have led to a digital transformation of the healthcare industry. Digital healthcare services have paved the way for easier and more accessible treatment, thus making our lives far more comfortable. However, the present day healthcare industry has also become the main victim of external as well as internal attacks. Data breaches are not just a concern and complication for security experts; they also affect clients, stakeholders, organizations, and businesses. Though the data breaches are of different types, their impact is almost always the same. This study provides insights into the various categories of data breaches faced by different organizations. The main objective is to do an in-depth analysis of healthcare data breaches and draw inferences from them, thereby using the findings to improve healthcare data confidentiality. The study found that hacking/IT incidents are the most prevalent forms of attack behind healthcare data breaches, followed by unauthorized internal disclosures. The frequency of healthcare data breaches, magnitude of exposed records, and financial losses due to breached records are increasing rapidly. Data from the healthcare industry is regarded as being highly valuable. This has become a major lure for the misappropriation and pilferage of healthcare data. Addressing this anomaly, the present study employs the simple moving average method and the simple exponential soothing method of time series analysis to examine the trend of healthcare data breaches and their cost. Of the two methods, the simple moving average method provided more reliable forecasting results.

## 1. Introduction

Advances in information and communication technology have helped the healthcare industry to replace paper-based systems with electronic health record (EHRs) systems to provide better and more cost-effective services to its customers. EHRs enhance patient care, develop patient cooperation, enhance disease diagnosis, improve practice efficiency, and make patient health information accessible all the time [1]. Additionally, smartphones and other web-based smart devices have changed the way we communicate. These devices empower users to easily and conveniently access the online services provided by different organizations. Healthcare is one among them. The last few years have seen healthcare data become more digitized, distributed, and mobile [2]. The Internet of Medical Things (IOMT) has also played a vital role in this context. Sensitive data are collected by healthcare organizations from their customers and stored on network servers to make them accessible all the time, and to facilitate patient care, but unfortunately, every blessing has a curse, which also applies here. The use of smartphones and other smart devices has also become a key source of privacy breaches [3]. Due to software vulnerabilities, security failures, and human error, these databases are sometimes accessed by unauthorized users. This leads to the exposure of sensitive data in the form of data breaches. Sometimes, insider attackers cause damage to protected health information, which results in the loss, theft, or disclosure of sensitive healthcare data. The price of a complete record file of a single patient can be hundreds of dollars on the dark web [4]. In comparison to other data industries, the healthcare industry is among the worst affected [5].

As reported by many practitioners, from 2005 to 2019, the total number of individuals affected by healthcare data breaches was 249.09 million. Out of these, 157.40 million individuals were affected in the last five years alone [6]. In the year 2018, the number of data breaches reported was 2216 from 65 countries. Out of these, the healthcare industry faced 536 breaches. This implies that the healthcare industry has faced the highest number of breaches among all industries [7]. There were 2013 data breaches reported from 86 countries in the year 2019 [8]. The total number of healthcare records that were exposed, stolen, or illegally disclosed in the year 2019 was 41.2 million in 505 healthcare data breaches [8]. According to an IBM report, the average cost of a data breach in 2019 was $3.92 million, while a healthcare industry breach typically costs $6.45 million [9]. This cost was the highest in the USA compared to other countries. Usually, a data breach would fetch $8.19 million. However, the average cost of a healthcare data breach (average breach size 25,575 records) in the USA is $15 million [10]. The average cost of a data breach increased by 12% from 2014 to 2019, and the average cost of a breached record increased 3.4% in the same time period. Moreover, the cost of a breached record in the healthcare sector registered an increase of 19.4%, the highest in this time period [10,11,12,13].

The aforementioned facts and figures show that the data assets of individuals and organizations are at risk. Even more alarmingly, the healthcare industry in particular is being targeted by attackers, and is therefore the most vulnerable. Thus, data privacy and confidentiality has become a serious concern for both individuals and organizations. Healthcare data are more sensitive than other types of data because any data tampering can lead to faulty treatment, with fatal and irreversible losses to patients. Hence, healthcare data need enhanced security, and should be breach-proof. In this study, our main concern was to investigate the healthcare data breaches reported or published by different eminent and authentic sources. We aimed to examine the causes of these breaches and use the results to improve healthcare data confidentiality. The analyzed factors that lead to healthcare data breaches will be addressed in our future research work to improve healthcare data confidentiality.

The rest of this study is divided into the following sections. The Section 2 defines the adopted methodology. The Section 3 provides information about the data sources. The Section 4 frames the analysis of data breaches, providing insights into the data breaches which are pertinent to healthcare. The Section 5 depicts the forecasting of healthcare data breaches. The Section 6 provides a discussion and the summarized results of this work, and the Section 7 chronicles the conclusion of the work.

## 2. Adopted Methodology

The sole aim of this study was to examine and investigate healthcare data breaches. This investigation was intended to provide insights into the causes and consequences of these occurrences on individuals and organizations. To this end, the authors analyzed different eminent and authentic data sources that included the Privacy Rights Clearinghouse (PRC), Health Insurance Portability and Accountability Act (HIPAA) journals, the Office for Civil Rights (OCR) Department of Health and Human Services (HSS.Gov.) USA, Ponemon Institute reports on data breach costs, and Verizon Data Breach Investigations Reports (Verizon-DBIR). In the next section, we briefly discuss these sources. The format of the data analysis method that was adopted in this study can be enumerated in the following steps:

- First, data are compiled from the sources mentioned above and presented in tabular form.
- Second, then sum, percentage, and average methods are applied to this data, and different types of patterns are extracted.
- Third, these patterns will help us to understand the sources and consequences of healthcare data breaches, the rise and downfall of data breaches, the behavior of different types of attacks, and other important things that are discussed in analysis section of this study.
- Fourth, a time series analysis is applied for healthcare data breach forecasting.

## 3. Data Sources

The data for the present research endeavor was obtained from the following sources:

PRC Database: PRC is a US based, non-profit organization established by Beth Givens in 1992. Its main purpose is to protect consumer information, to provide consumer advocacy services and guidelines to control personal information, and to improve consumer awareness about the technological effects of personal privacy. It provides a complete database of data breaches. The database has a record of 9016 data breach instances reported by different organizations. According to the PRC database, more than 10 billion user records have been compromised since 2005.

HIPAA Journal: The HIPAA journal is an effective outcome of the HIPAA Act 1996. It is a US-based journal that provides comprehensive information about healthcare data breaches, guidelines for HIPAA compliance, and practical guidelines for data breach avoidance. It has been providing comprehensive information about healthcare data breaches since September 2009.

OCR Reports: The Office for Civil Rights Department of Health and Human Services of the USA also provides yearly/bi-yearly or tri-yearly data breach reports, named, “Report to Congress on Breaches of Unsecured Protected Health Information”. These reports provide comprehensive information about healthcare data breaches from 2009 to 2017 [14,15].

Ponemon Institute Reports: The Ponemon Institute is an eminent research institute that mainly focuses on the protection of data, privacy, and security of information issues and policies. It was established in 2002 in Michigan by Dr. L. Ponemon. The institute’s reports are a repository of authentic records on data breach costs, sponsored by IBM.

Verizon-DBIR: Data breach investigation reports by Verizon Enterprises comprise yearly investigations reports on data breaches. The first such report was published in 2008. The reports record instances of data invasion in private as well as public organizations across the world.

All these are globally accepted sources of eminent and authentic data on data breaches.

For this research endeavor, we have premised our analysis on the sources that are mentioned above to examine healthcare data breaches and their causes and consequences. These sources have enabled us to garner an in-depth understanding of patterns in data breaches, and have facilitated our research on mapping the implications.

## 4. Analysis of Data Breaches

Generally, a data breach is an illegal disclosure or use of information without authorization. The United States Department of Health and Human Services defines a data breach as “the illegal use or disclosure of confidential health information that compromises the privacy or security of it under the privacy rule that poses a sufficient risk of financial, reputational, or other type of harm to the affected person” [11]. The HIPAA definition of a data breach is “the procurement, access, use or expose of confidential health information illegitimately, which compromises the privacy or security of that confidential health information” [14].

Data breaches can harm individuals and organizations in several ways. Besides the huge financial setback that organizations have to deal with in cases of data pilferage, such instances also dent the image of the organizations, marring their reputation and brand value. Data breaches are usually classified into two major categories: internal and external. Internal data breaches comprise incidents that are occur with the help of an internal agent. These may be privilege abuse, inauthentic access/disclosure, improper disposal of unnecessary but sensitive data, loss or theft, or the unintentional sharing of confidential data to an unauthorized party. External data breaches are incidents caused by any external entity or source. These include any hacking/IT incident such as a malware attack, ransomware attack, phishing, spyware, or fraud in the form of stolen cards, etc.

The Privacy Rights Clearinghouse (PRC), a nonprofit organization based in the USA, reported that there were 9016 data breach instances in different sectors from January 2005 to October 2019. The total number of records exposed in these breaches was more than 10 billion (10,376,741,867) [6]. The different types of attacks used to breach the information were Intentional Insider Attacks (INSD), Frauds Using Cards (CARD), Physical Damage such as the theft or loss of paper documents (PHYS), Damage of Portable Device such as lost or theft (PORT), Hacking or Malicious Attacks (HACK), Stationary Computer Loss (STAT), Unknown Approaches (UNKN), and Unintentional Disclosure (DISC). The organizations that were affected by these data breaches may be classified into the following categories:

Some data breach incidents corresponding to each sector have been reported in the PRC database. Since in these intrusions, no records were breached, the authors have not included those numbers in their reference on the representation of data breaches by sector. After an exhaustive analysis of the PRC database, the compiled information was tabulated in Table 1.

Table 1 presents information on data breach incidents by sector in two scenarios. The first scenario is a collation of the breach episodes that have occurred in the last 15 years. The second scenario, which is the core focus of our study, records the breach episodes that occurred in the healthcare industry in the last 5 years. A comparative analysis of the two scenarios clearly reveals that the healthcare industry is most susceptible to data pilfering.

A thorough analysis of the entire 15-year timeframe shows that the healthcare (MED) sector in both the time frames from (2005 to 2019) and (2015 to 2019) has faced the highest number of data breaches. Out of the 6355 breach incidents reported during 2005–2019, 3912 were recorded in the healthcare industry alone. This accounts for 61.55% of the total. The MED sector is followed by the EDU and GOV sectors, which account for 10.55% and 8.82%, respectively. Out of the 3912 incidents that the healthcare industry faced, 1587 were carried out in the last five years (2015 to 2019), comprising 40.56% of the total healthcare data breaches. This is cause for great alarm, and calls for immediate remedial action.

In the second case, from 2015 to 2019, there were a total of 2027 data breach incidents faced among the specified sectors. Out of these 2079 incidents, 1587 were recorded in the healthcare (MED) sector, which is 76.59% of the total. The MED sector is followed by the BSF sector, with a share of 9.36%. However, the other sectors show a small decrease in incidents. The data clearly shows that the healthcare industry has become the main victim of data breaches. Moreover, the rate of healthcare data breaches has increased even more rapidly in the last five years.

**Figure 1:**
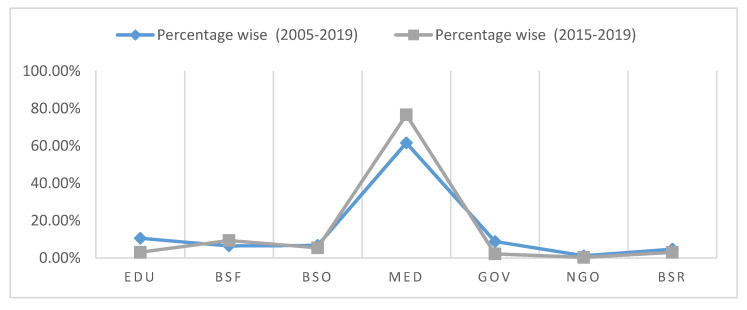
Representation of Data Breach Incidents.

Figure 1 presents a graphical representation of Table 1. The figure shows that the slope of the graph in each sector has witnessed a decrease in the second case (2015–2019), except in the MED sector, followed by the BSF sector. The graph indicates that the healthcare industry is the preferred target of attackers because of the high commercial value of EHRs.

### 4.1. Healthcare Data Breach Analysis

Generally, healthcare data breaches can be defined as “illegitimate access or disclosure of the protected health information that compromises the privacy and security of it”. To analyze healthcare data breaches, the authors investigated the MED domain of the PRC database thoroughly [6]. Table 2 provides information about healthcare data breaches reported by PRC. In this table, the data has been presented in two different scenarios. In the first scenario, data is presented as whole from 2005 to 2019. In the second scenario, we presented the data in three clusters, i.e., from 2005 to 2009, 2010 to 2014, and 2015 to 2019. The objective of data clustering is to detect changes in the trends of healthcare data breaches with the passage of time.

Analysis of Table 2 shows that 249.09 million people were the victims of healthcare data breach episodes. From 2005 to 2009, 13.49 million Health Records were exposed, i.e., 5.41% of the total number of cases. In the period from 2010 to 2014, 78.18 million records were exposed; this makes up 31.38% of the total. From 2015 to 2019, 157.40 million records were exposed, that is, 63.19% of the total. In addition, out of 249.09 million records, 161.05 were exposed through hacking attacks that comprised 64.65% of the total exposed health records from 2005 to 2019. An interesting pattern that can be detected here is that:

- In first cluster of five years (2005 to 2009), only 0.6 million records were exposed through hacking.
- In the second cluster of five years (2010–2014), 14.70 million records were exposed through hacking.
- In the third cluster of five years (2015–2019), 145.75 million records were exposed.

Thus, it is evident that the healthcare industry has been inundated by hackers in the last five years, compromising 90.49% of health records during this time period.

This analysis places the healthcare industry in a very vulnerable position. Yet another facet to note is the types of attacks employed for data breaches. Other than HACK attacks, the healthcare sector has also been targeted by PHYS and PORT attacks. Reports state that 14.39% of PHYS attacks and 9.51% of PORT attacks were engineered from 2005 to 2019. But in last five years (2015 to 2019), a significant decline has been recorded in the numbers of HACK and PHYS attacks. Only 1.75% of reported attacks from 2015–2019 were HACK, and as few as 0.25% were PHYS. The highest number of data breaches from 2005 to 2019 was in the form of DISK type attacks. There were as many as 1019 DISK attacks out of a total of 3912 data breach incidents, comprising 26.04% of the total. However, these attacks only succeeded in exposing 13.77 million records.

**Figure 2:**
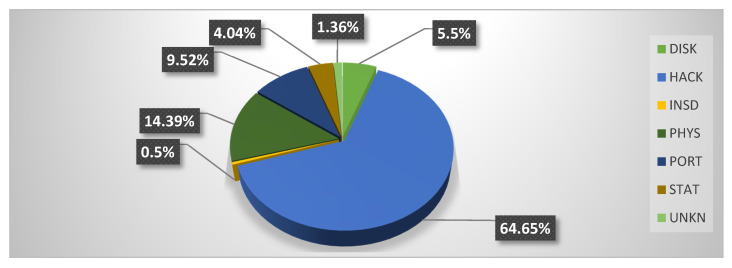
Proportion of Records Exposed From 2005–2019 with Different Types of Attack.

**Figure 3:**
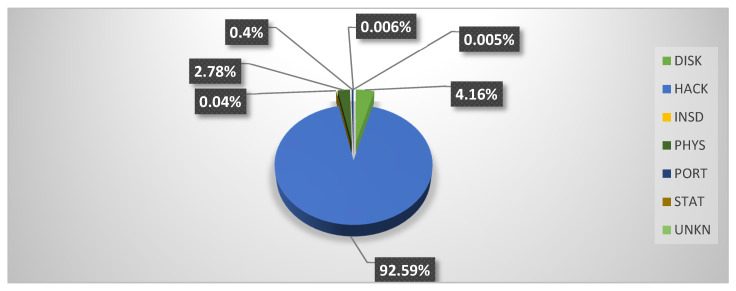
Proportion of Records Exposed from 2015–2019 with Different Types of Attack.

Figure 2 and Figure 3 depict the proportion of records exposed with each type of attack, given in percentages, from 2005 to 2019 and 2015 to 2019, respectively. Both figures show that hacking is the main cause behind the exposure of highly sensitive health records. The figure also shows an abrupt increase in hacking incidents in the same time zone.

Furthermore, Figure 2 and Figure 3 show that the INSD (Intentional Insider Attacks) and UNKN (Unknown Approach)-type attacks have the least effect on the healthcare industry. INSD and UNKN were responsible for only 0.5% and 1.36%, respectively, of the total number of exposed records from 2005 to 2019. The type of attacks that have shown a rapid decrease in the last five years (2015–2019) are PHYS, from 14.39% to 2.78%, PORT, from 9.52% to 0.4%, and STAT, (Stationary Computer Loss) from 4.04% to 0.0006%.

CARD (Fraud involving Debit and Credit Cards) is a type of attack mentioned in the OCR database specifications, but we could not confirm any such data breaches. Hence, we have not included CARD in our analysis. Cyber-attacks are carried out to disrupt computer server systems, and in our study, we have bracketed them under the umbrella of Hacking/IT incidents.

### 4.2. HIPAA and OCR Data Breach Report Analysis

The authors of this study have also compiled the data of healthcare breaches published by the HIPAA journal from 2010 to 2019. The data were outsourced and analyzed from different monthly, yearly, and other reports published by HIPAA. It is not possible to provide references of every HIPAA journal report that we referred to in compiling the data; therefore, we have only cited the main references of the journal reports. These references authenticate our data. The data are presented in Table 3 [8,16,17]. We found quantitative variations in some reports while compiling the same data, e.g., the number of data breaches reported in 2014 was 307 in one HIPAA report and 314 in another. In such contradictory cases, we opted to take the data from the latest report.

In Table 4, we present the compiled data of different reports generated by the Office for Civil Rights with the title name “Report to Congress on Breaches of Unsecured Protected Health Information” from 2010 to 2017. The OCR data breach reports for the years 2018 and 2019 have not been published by the OCR yet [15].

To check the accuracy and consistency of the data, we compared it only with the compiled data of HIPAA and OCR reports from 2009 to 2017 because of the unavailability of OCR data for 2018–2019. A comparative study of the HIPAA and OCR data breach reports shows a small variation in number of breaches recorded each year and the number of exposed records from these breaches. The total number of breaches reported by HIPAA from 2010 to 2017 was 2163, and the total number of records exposed from these breaches was 180.65 million, while the total number of breaches reported by the OCR for the same period was 2244, and the total number of records exposed from these breaches was 180.6 million. The HIPPA and OCR data note that the highest number of data breaches was reported in 2017, whereas the highest number of records was exposed in 2015.

The data analysis in Table 3 and Table 4 shows that the healthcare sector saw a mercurial rise in data breach cases in 2015, when more than 40% of the health records were exposed. After 2015, the maximum number of health records was exposed in the 2019. The number of cases accounted for 16.14% of the total of 255.18 million exposed health records from 2010 to 2019. The compiled data also shows that the number of healthcare data breach cases was considerably less in the 2017, when only 5.1 or 5.7 million records were breached. An overall analysis indicates that the data breach trend started to show an abrupt increase from the year 2014.

#### 4.2.1. Data Disclosure Types

A comprehensive analysis was carried out on HIPAA data breach reports. It was found that the main disclosure types of protected healthcare information were hacking incidents, unauthorized access (internal), theft or loss, and improper disposal of unnecessary data. The procedure that we discussed in Section 4.2 for references [8,17,18] is also followed in this context. The different disclosure types mentioned above are briefly explained below:

Hacking Incidents: Hacking incidents comprise all cyber-attacks that are used to gain unauthorized access to confidential data. Ransomware and malware are the main approaches that are used to expose protected health information [8,17].

Unauthorized Access (internal): These includes all types of attacks that lead to the exposure of confidential health data with the help of any internal source of an organization. This may be abuse of privileges, unauthenticated access/disclosure, etc.

Theft or loss: This comprises all incidents that lead to the disclosure of protected health information in the form theft or loss, such as the theft of hard disks, laptops, or any other portable device that contains protected healthcare data. This can also be because of catastrophic damage or the loss of these devices.

Improper disposal of unnecessary data: Unnecessary but sensitive and confidential data should be properly disposed of so that it cannot later be retrieved. Improper disposal of this data can lead to the disclosure of protected health information. Improper disposal attack type includes all breached incidents that are caused by the improper disposal of unnecessary but sensitive and confidential health data.

Table 5 presents detailed information about the number of healthcare data breach incidents carried out with these disclosure types.

In this table, we present the number of breach incidents executed by a particular disclosure type from 2010 to 2019. As per the table, the following facts can be underscored:

- From 2010 to 2019, a total of 2860 breached incidents were carried out through the aforementioned disclosure types.
- 29.72% of breach instances were due to separately hacking/ IT incidents.
- 29.47% of breach instances were due to internal unauthorized disclosures.
- 37.65% of instances were due to theft/loss cases.
- 3.14% of instances occurred due to the improper disposal of unnecessary but sensitive data.
- The overall results show that theft/loss cases are the highest in number, followed by Hacking/IT incidents and unauthorized internal disclosure, while there are very few cases of improper disposal in the ten-year period.
- When we analyzed the pattern over the last four years, we found an abrupt increase in hacking/IT incidents. Out of the 850 hacking/IT incidents reported in ten years (2010–2019) period, 692 incidents were reported in the last four years alone (2016–2019); that accounts for 81.85% of the total, among which 32.23% were reported in 2019 alone.

Thus, this analysis clearly depicts that hacking and other IT-related attacks have become a serious concern for the healthcare data industry. Unauthorized access/ internal disclosure have also shown an increase in the last few years, but not as fast as hacking incidents. Out of the total of 843 unauthorized internal disclosure incidents, 542 were reported in the last four years. This figure comprises 64.29% of the total, and out of this, 16.84% incidents were reported in 2019. A comparison of this proportion (16.84%) with last year (2019) shows that hacking incidents increased by 32.23%. This is double the number of unauthorized internal disclosure incidents. Here, we also found how hacking incidents became more frequent and became a severe concern for the healthcare sector.

**Figure 4:**
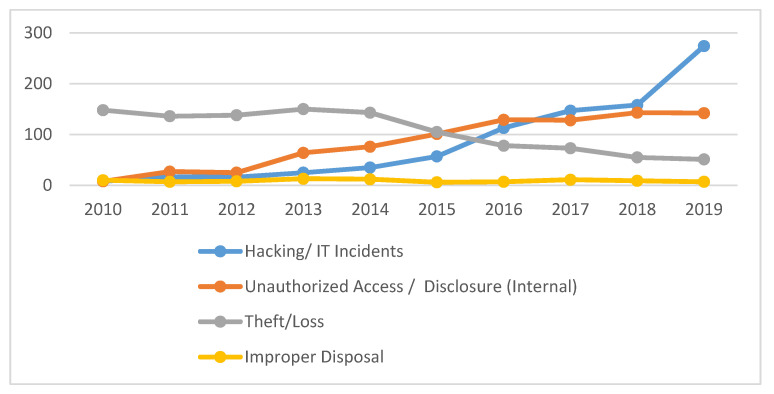
Graphical Presentation of Different Data Disclosure Types.

On the other hand, theft/loss and improper disposal have shown a clear decrease in the last four years. Out of a total of 1077 theft/loss incidents, only 257 were reported in the last four years, that is, 23.86% of the total. Furthermore, out of a total of 90 improper disposal cases, only 34 were reported in last four years, that is, 37.77% of the total. These calculations show that theft/loss and improper disposal have a far less adverse effect on the healthcare industry. Figure 4 provides a graphical presentation of different disclosure types.

The above graph shows that theft/loss and improper disposal incidents have decreased in frequency, but that hacking/IT incidents and unauthorized access incidents have increased. Notably, hacking/IT incidents have shown an abrupt increase over the last few years. In the next subsection, we will discuss the locations of breached information and from where the sensitive health information has been breached/disclosed.

#### 4.2.2. Breached Locations

Protected health information is stored either on paper or on electromechanical storage devices. This section details the locations from where the protected health information is breached through different approaches. Yearly information about the location of data breach incidents is shown in Table 6. The data presented in this table were compiled from OCR and HIPAA reports. In this context, we also followed the same procedure as discussed in Section 4.2. We have provided only the important references [8,13,17,18,19,20,21], because for 2018 and 2019, we referred to 24 different reports, and including all of them in this study would not be feasible.

In Table 6, eight locations, i.e., Electronic Medical Records (EMR), Laptop, Desktop computers, Other Portable electronic devices, Paper documents, Network Server, Email, and Other, are the locations from where the protected health information (PHI) was breached. According to the analysis, out of the 8 locations, Paper/Film is the most susceptible to breaches. It saw 575 breached incidents out of a total of 3253 incidents, accounting for 17.67% of the total number of episodes during 2010 to 2019. The leading position of Paper/Films is because of the improper disposal of unnecessary but sensitive healthcare data. Paper/Films is followed by Email, which represented 17.52%, and Network servers, which accounted for16.69% of the total.

Electronic Medical Records (EMR) saw the least fewest instances of intrusion, with only 195; this is only 5.99% of the total of 3253 incidents carried out in the same time period. EMR is followed by the Other Portable Electronic Devices (PED) which made up 6.64% of the total. Desktop computers accounted for 9.40% of the total. As per the data, the attacks on Email and Network Server locations showed a marked increase from 2016–2019. Out of a total of 570 Email location based data breach incidents, 457 were reported in the last four years (2016 to 2019), of which 35.03% were reported in the year 2019 only. Moreover, out of a total of 543 Network server location-based data breach incidents, 348 were reported in the last four years (2016 to 2019). Yet again, 22.03% of these cases were reported in 2019 alone. This is because of the digitization of healthcare organizations and the excessive use of smart devices by customers. Studies also show that outdated security software, Database servers without passwords, and email accounts with weak or no passwords are the most common reasons behind these breaches. Our analysis also revealed that Paper/Films, Desktop computers, and Laptops have shown a small decrease in the number of breaches over the last four years.

**Figure 5:**
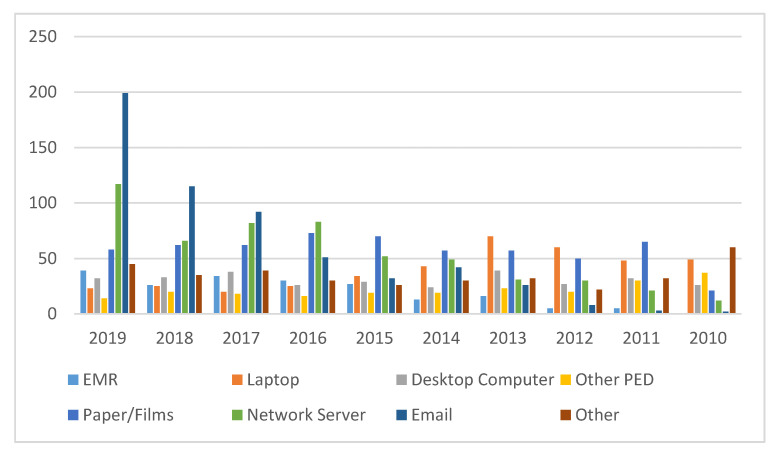
Comparative Graphical Representation.

Our study observed that at present, attacks on sensitive healthcare data are being perpetrated by cyber criminals who use different techniques such as malware, ransomware, or phishing attacks [8,17] to prey on EHRs. Email and Network servers have become attack-prone locations for hackers. Figure 5 shows a comparative representation of these locations on the basis of the number of breached incidents every year carried out on each location. Graphical representation will help the reader to understand the results that we have produced through this analysis, and will also help to map the variation of healthcare data breach incidents carried out on specified locations over a ten-year period.

### 4.3. Financial Effect of Data Breaches

Data breach cost calculation is a complex task. Different institutions have set parameters and applied different techniques to estimate the average cost of data breaches. The Ponemon Institute calculates both direct and indirect expenses incurred by an organization to determine the average cost of a data breach. This section discusses the financial effects of data breaches, and mainly focuses on healthcare data breaches. For this purpose, the data breach cost reports generated by the Ponemon Institute sponsored by IBM were analyzed to determine the financial effects of data breaches on individuals, organizations, and countries. Table 7 provides information about data breach costs from 2010 to 2019. The data presented in this table were compiled from different Ponemon-IBM sponsored data breach cost reports [12,13,22,23,24,25,26,27].

**Figure 6:**
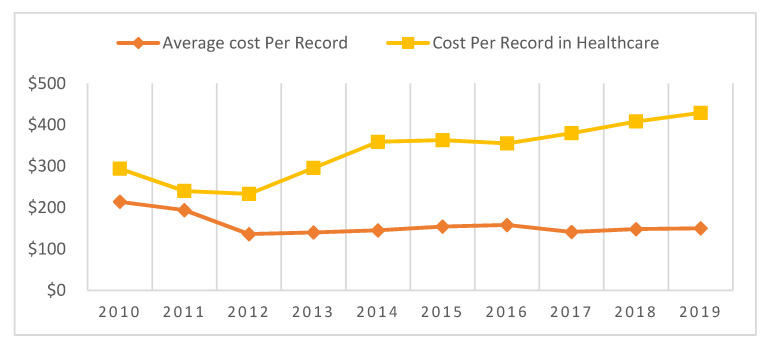
Graphical Comparison of Average Record Cost and Healthcare Record Cost.

Data breach cost analysis shows that healthcare breached record costs have increased rapidly compared to the average cost of a breached record. The average record cost was $214 in 2010, but in 2011, it had decreased by 10%. In 2012, it decreased by 42.64% from the previous year. After that, it gradually increased or decreased year by year; in 2019, it increased by 1.55% from the previous year. From 2010 to 2019, the healthcare breached record cost increased by 45.91% from $294 to $429. The cost of each breached record in the healthcare sector was $294 in 2010; this figure decreased until 2012, after which it increased by 1.11% from 2014 to 2015, 7.04% from 2016 to 2017, and 5.14% from 2018 to 2019. The 2018 Verizon DBIR report showed that 76% of data breaches carried out in 2018 were financially motivated [7]. In line with that report, it was shown that 83% of healthcare data breaches had financial motives [21]. Figure 6 provides a graphical cost comparison of average breached record costs and healthcare breached record costs by year. In the next subsection of this study, we will perform a time series analysis to find the trend of healthcare data breaches and their costs.

## 5. Forecasting of Healthcare Data Breaches

The time series analysis is a statistical approach that is used for forecasting or trend analysis; it works on data sets ordered in time, or deals with time series data. Time series data defines the set of values that a variable takes at different times. In this study, the Simple Moving Average (SMA) and Simple Exponential Smoothing (SES) methods of time series were applied to the data to determine the trend of healthcare data breaches and their cost on the healthcare industry. Two methods of time series were applied to the same data in this study to determine the variations, if any, and to make the forecasting results more consistent.

The Simple Moving Average (SMA) method has been adopted extensively to keep update forecasts. This method is based on the calculated averages of subsets of a data set. The moving average can be calculated by making subgroups of observations. It can include two, three, four, or five observation groups. After calculating the moving average, it was used to forecast the next period [25].

At = (Ot + Ot-1 + … + Ot-n + 1)/n(1)

where At is moving average at time t, which is the forecast value at time t + 1; Ot is Observation at time t; and ‘n’ is number of observations in an interval or sub-group [28].

**Figure 7:**
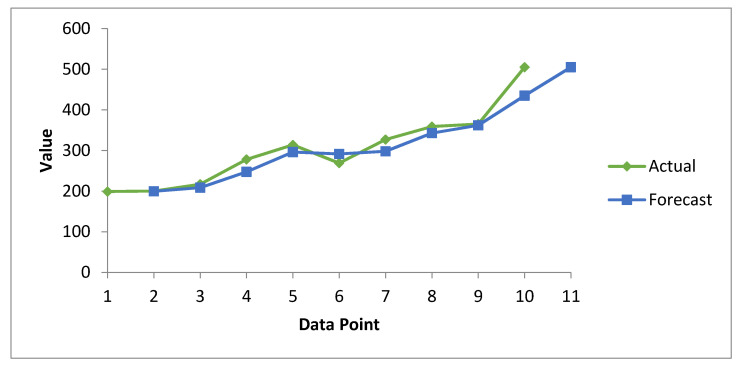
Forecasting Graph of Healthcare Data Breaches from 2010–2020 through SMA method.

**Figure 8:**
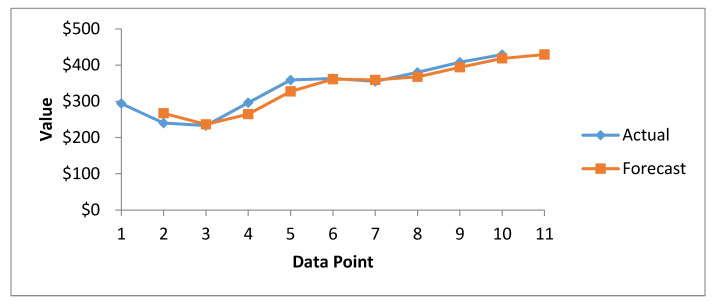
Forecasting graph of Healthcare Record Cost since 2010–2020 through SMA method.

Here we take the interval of two observations as a subgroup, and the moving averages are calculated. Table 8 summarizes the forecast information about healthcare data breaches via the SMA method. Here, the actual values represent known observations, while the forecast values are the predicted values calculated using the SMA method. With the help of the data analysis tool in Microsoft excel, we generated the forecast results and compared them with manually calculated results with the help of Equation (1) for accuracy. In the interests of brevity, we have only showed the final forecast results in tabular and graphical form. Figure 7 provide a graphical presentation of the forecast data breaches, while Figure 8 cites the forecast costs for breached healthcare records.

In Figure 7, the green curve represents actual data breaches, while blue represents forecast data breaches calculated on the basis of the moving average. Both curves are close to each other and show an increasing trend. However, the actual curve always lies above the forecast line, which predicts that the magnitude of data breaches will increase in the coming years. Hence, all necessary and preventive measures have to be taken by researchers, security experts, and healthcare organizations to minimize this.

Figure 8 presents the results of cost forecasting of exposed health records, determined using the SMA method. The actual and forecast curves are close to each other from the beginning to the end. However, the actual curve consistently grows from the upper side. The forecast curve shows that the cost of healthcare breached records increases consistently. From this trend, we can predict that in future, the cost of healthcare breached records will increase, albeit gradually.

Simple Exponential Smoothing (SES) is a forecasting method used for univariate data. It is one of the most popular forecasting methods that uses the weighted moving average of past data as the basis for a forecast. Unlike the Simple Moving Average method, the idea of this method is to provide the highest weights to recent data points (observations) and the lowest weights to older data points (observations) [29]. It is better known for short-term forecasting, and its accuracy strongly depends on the optimal value of the smoothing constant, α. The value of α is between 0 and 1. When α is close to 1, fast learning is indicated (in this case, forecasting is influenced by only the most recent values), whereas when α is close to 0, slow learning (in that case, forecasting is influenced by old observations) occurs. The general formula for SES is:

F_t+1_ = α y_t_ + (1 − α) F_t_ (2)

where F_t+1_ is the forecast value at time t + 1, α is the smoothing constant, y_t_ is a known value at time t, and Ft is the forecast value of the variable Y at the time t [29]. Here, we take the value of α = 0.4 so as to accomplish a balanced influence of observations on the forecasting results. Table 9 provides the forecast results of healthcare data breaches and their cost, determined using the SES method. The forecast values were calculated on the basis of actual (known observations) values using Equation (2). Later, we compared the results with those generated by the data analysis tool in MS-Excel to verify the accuracy. The final results of the forecasting are presented in Table 9.

**Figure 9:**
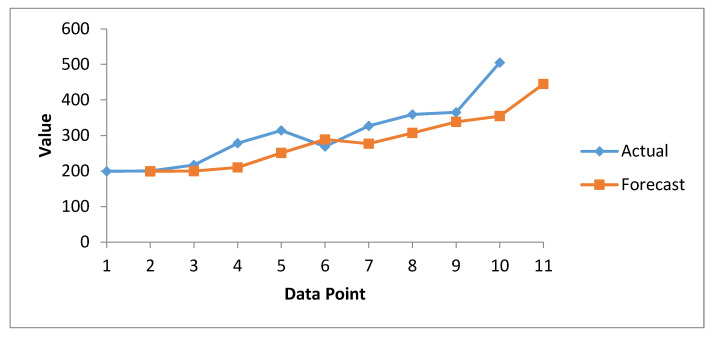
Forecasting Graph of Healthcare Data Breaches from 2010–2020 using the SES method.

Figure 9 summarizes healthcare data breach forecasting using the SES method. The actual curve of the graph always moves above the forecast curve except at data point 6, where the forecast data point value is higher than the actual (known) value. For the year 2020, only a forecast value was available, which we predicted on the basis of previous historical data.

**Figure 10:**
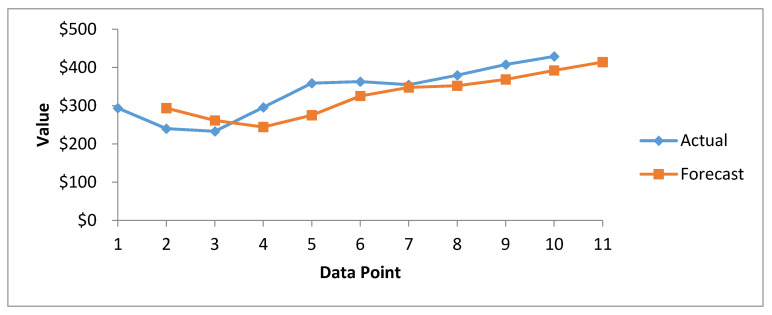
Forecasting graph of Healthcare Record Costs from 2010–2020 Using the SES method.

Figure 10 presents the results of cost forecasting of exposed health records, as determined using the SES method. The data points on the actual curve represent the original values of observations, whereas those on the forecast curve represent prediction (forecasting) values.

## 6. Discussion

The transformation of the healthcare industry from one that uses paper-based systems to one that is based upon electronic health record systems has been made possible because of smart phones, information systems, IOMT, cloud services, internet connectivity, and other web based smart devices. Advances in information and communication technology have made healthcare data more digitized, distributive, and mobile. Despite the numerous advantages of EHRs, the digital health data of patients is at huge risk today. As chronicled in our study, data breach trends have also undergone a massive transformation. The comprehensive analysis undertaken in this study reveals that the healthcare industry is the focus of many cyber invaders. Moreover, we analyzed different data breach reports generated by different organizations and institutes to gain insights, and apply them, in our future research work. The final results which from this study are:

- More than 10 billion records were exposed from different sectors from 2005 to 2019. These sectors were MED, BSF, BSO, EDU, NGO, BSR, and GOV.
- There have been 3912 confirmed data breach cases in the healthcare sector alone. Nearly 43.38% of health data was compromised from 2005 to 2019, the highest among all sectors.
- The greatest number of breach attacks on EHRs was initiated by Hacking (HACK). Statistics show that more than 64% of health data was breached from 2005–2019. Moreover, in the last five years (2015–2019) alone, hacking incidents exposed more than 92% of records. This shows an alarming change in hacking attacks on healthcare organizations. Other types of attacks that affected the healthcare industry were PHYS and PORT, being the causes of 14.39% and 9.51% of the total exposed records from 2005 to 2019, respectively.
- HIPAA and OCR reports also showed that hacking/IT incidents are the main cause behind healthcare data breaches. As per the HIPAA reports, 255.18 million people were affected from 3051 healthcare data breach incidents from 2010 to 2019.
- The main types of attacks used to breach protected health data are Hacking/IT incidents, unauthorized access/ internal disclosure, Theft/loss, or Improper disposal. However, in the last three or four years, theft/loss and improper disposal have shown a decreasing trend. In contrast, hacking/IT incidents and unauthorized internal disclosures have shown a marked increases, especially hacking incidents, which have increased very rapidly in frequency in last few years.
- Hacking/IT incidents have increased by 73.4% in 2019 from 2018. However, unauthorized internal disclosure, theft/loss, and improper disposal decreased by 0.7%, 7.8%, and 22.22%, respectively, from 2018 to 2019.
- The main locations from where confidential healthcare data were breached over the last four years were email and network servers. Paper/Films have also been major targets since 2010, although there has been a decrease in attacks on Paper/Films in the last four years.
- In the healthcare industry at present, the average cost of data breach is $6.45 million, up from $3.92 million in 2019 [9]. The average cost of a breached record is $150. But in the healthcare industry, the cost of each breached record was $429 in 2019 [13]. The average cost of each record increased by 1.35% in 2019 relative to 2018, and the cost of each breached record in the healthcare sector increased by 5.14% in 2019.
- The SMA and SES methods of time series analysis were used for healthcare data breach and cost forecasting. The generated results indicated that SMA provides more accurate forecast results than SES. SMA produced results which showed more symmetry with the actual results than the SES results.

## 7. Conclusions

From our analysis of healthcare data breaches, the authors concluded that E-health data is highly susceptible, as it is targeted most frequently by attackers. A long-term analysis of data breaches showed that healthcare records were exposed by both internal and external attacks, such as hacking, theft/loss, unauthentic internal disclosure, and the improper disposal of unnecessary but sensitive data. However, our short-term analysis showed that hacking/IT incidents are most commonly used by attackers. Furthermore, the short-term analysis also showed that Email and Network servers are the main locations from where confidential health data is beached. Our cost analysis showed that healthcare data breaches are far more expensive than the average cost of data breaches, especially in developed countries. The time series analysis results showed that both data breaches and their costs will increase in future. Hence, preventive measures need to be prioritized by the researchers, security experts, and healthcare organizations.

There are several other aspects that need to be focused upon in research that seeks to provide insights into healthcare data breaches. The authors of the present study only used the most pertinent ones. However, the authors intend to pursue the following specific domains in the future:

- Identify and address the main victims of cyber-attacks on the healthcare sector.
- Undertake a study that investigates whether healthcare organizations are lacking usable-security measures because of the absence of accountability and improper training of employees and clients.
- Classify hacking/IT incidents that led to healthcare data breaches.
- Identify preventive measures that should be taken to avoid healthcare data breaches.

## Figures and Tables

**Table 1:** Representation of Data Breaches by Sector.

Name of Sector	Data Breaches in Last 15 Years (2005–2019)		Data Breaches in Last 5 Years (2015–2019)	
	Number of Breaches	Percentage (%)	Number of Breaches	Percentage (%)
EDU	671	10.55	64	3.08
BSF	410	6.45	194	9.36
BSO	426	6.70	113	5.45
MED	3912	61.55	1587	76.59
GOV	561	8.82	45	2.17
NGO	75	1.18	7	0.33
BSR	300	4.72	62	2.99
Total	6355	99.97	2072	99.97

EDU: Educational Organizations; BSF: Businesses-Financial; BSO: Businesses-Other; BSR: Business-Retail Includes Online Retail; MED: Healthcare Service Providers; GOV: Government and Defense Institutes; NGO: Non-Governmental Organizations.

**Table 2:** Types of Attacks on MED Sector and Number of Individuals Affected.

Type of Attack	Scenario-I		Scenario-II					
	Number of Breaches	Individuals Affected in Millions	Number of Breaches			Individuals Affected in Millions		
	(2005–2019)	(2005–2019)	(2005–2009)	(2010–2014)	(2015–2019)	(2005–2009)	(2010–2014)	(2015–2019)
DISK	1019	13.71	28	406	585	0.75	6.41	6.55
HACK	806	161.05	8	241	557	0.60	14.70	145.75
INSD	181	1.24	21	146	14	0.24	0.93	0.07
PHYS	1315	35.85	33	905	375	0.14	31.33	4.38
PORT	382	23.71	94	238	51	11.05	12.02	0.64
STAT	86	10.08	14	72	1	0.44	9.64	0.0009
UNKN	123	3.42	4	115	4	0.27	3.15	0.0008
Total	3912	249.09	202	2123	1587	13.49	78.18	157.40

HACK: Hacking or Malicious Attacks; INSD: Intentional Insider Attacks; PHYS: Physical Damage such as the theft or loss of paper documents; PORT: Damage of Portable Device such as lost or theft; STAT: Stationary Computer Loss; UNKN: Unknown Approaches.

**Table 3:** HIPAA Reported Healthcare Data Breaches.

Year	Number of Data Breaches	Exposed Records in Millions
2010	199	5.530
2011	200	13.150
2012	217	2.800
2013	278	6.950
2014	314	17.450
2015	269	113.270
2016	327	16.400
2017	359	5.100
2018	365	33.200
2019	505	41.200
Total	3033	255.18

**Table 4:** Reported Healthcare Data Breaches.

Year	Number of Data Breaches	Individuals Affected in Millions
2010	207	5.400
2011	236	11.410
2012	222	3.270
2013	294	8.170
2014	277	21.340
2015	289	110.700
2016	334	14.570
2017	385	5.740
2018	−	−
2019	−	−
Total	2244	108.80

**Table 5:** Type of Healthcare Data Breaches.

Year	Disclosure Types			
	Hacking/IT Incidents	Unauthorized Access/Disclosure (Internal)	Theft/Loss	Improper Disposal
2010	8	8	148	10
2011	17	27	136	7
2012	16	25	138	8
2013	25	64	150	13
2014	35	76	143	12
2015	57	101	105	6
2016	113	129	78	7
2017	147	128	73	11
2018	158	143	55	9
2019	274	142	51	7
Total	850	843	1077	90

**Table 6:** PHI Breached Location.

Year	EMR	Laptop	Desktop Computer	Other PED	Paper/Films	Network Server	Email	Other	Total
2019	39	23	32	14	58	117	199	45	527
2018	26	25	33	20	62	66	115	35	382
2017	34	20	38	18	62	82	92	39	385
2016	30	25	26	16	73	83	51	30	334
2015	27	34	29	19	70	52	32	26	289
2014	13	43	24	19	57	49	42	30	277
2013	16	70	39	23	57	31	26	32	294
2012	5	60	27	20	50	30	8	22	222
2011	5	48	32	30	65	21	3	32	236
2010	0	49	26	37	21	12	2	60	207
Total	195	397	306	216	575	543	570	351	3253

PHI: Protected Health Information; EMR: Electronic Medical Records; PED: Portable Electronic Devices.

**Table 7:** Cost of Data Breaches.

Year	Average Cost of Breach in Millions	Average Cost Per Record	Cost Per Record in Healthcare
2010	$7.24	$214	$294
2011	$5.50	$194	$240
2012	$3.20	$136	$233
2013	$3.29	$140	$296
2014	$3.50	$145	$359
2015	$3.79	$154	$363
2016	$4.00	$158	$355
2017	$3.62	$141	$380
2018	$3.86	$148	$408
2019	$3.92	$150	$429

**Table 8:** Healthcare Data Breach Forecasting through SMA method.

Year	Number of Data Breaches (Actual Values)	Forecast Values by SMA Method	Cost Per Record in Healthcare (Actual Values)	Forecast Values by SMA Method
2010	199	N/A	$294	N/A
2011	200	199.5	$240	$267
2012	217	208.5	$233	$237
2013	278	247.5	$296	$265
2014	314	296	$359	$328
2015	269	291.5	$363	$361
2016	327	298	$355	$359
2017	359	343	$380	$368
2018	365	362	$408	$394
2019	505	435	$429	$419
2020	−	505	−	$429

SMA: Simple Moving Average.

**Table 9:** Healthcare Data Breach Forecasting Using the SES method.

Year	Number of Data Breaches (Actual Values)	Forecast Values by SES Method	Cost Per Record in Healthcare (Actual Values)	Forecast Values by SES Method
2010	199	N/A	$294	N/A
2011	200	199	$240	$294.00
2012	217	199.6	$233	$261.60
2013	278	210.04	$296	$244.44
2014	314	250.816	$359	$275.37
2015	269	288.7264	$363	$325.55
2016	327	276.8906	$355	$348.02
2017	359	306.9562	$380	$352.20
2018	365	338.1825	$408	$368.88
2019	505	354.273	$429	$392.35
2020	−	444.7092	−	$414.34

SES: Simple Exponential Smoothing.
